# Decreased expression of LEF1 caused defective decidualization by inhibiting IL-11 expression in patients with adenomyosis

**DOI:** 10.1186/s10020-024-01054-9

**Published:** 2025-01-10

**Authors:** Jingru Duan, Xiaowei Zhou, Hanfei Zhu, Mingjuan Zhou, Mengyu Liu, Yan Zhou, Wenzhu Li, Bufang Xu, Aijun Zhang

**Affiliations:** 1 Department of Obstetrics and Gynecology, Ruijin Hospital, Shanghai Jiao Tong University School of Medicine, Shanghai, China; 2 Shanghai Key Laboratory for Assisted Reproduction and Reproductive Genetics, Shanghai, China

**Keywords:** Adenomyosis, LEF1, IL-11, Endometrial receptivity, Endometrial decidualization

## Abstract

Reduced lymphoid enhancer-binding factor 1 (LEF1) expression in patients with adenomyosis during the mid-secretory phase leads to impaired endometrial receptivity, affecting embryo implantation. This study investigated the molecular mechanisms underlying reduced endometrial receptivity in 25 adenomyosis patients and 25 controls. Functional experiments were conducted using human endometrial stromal cells (HESCs) and TERT-immortalized HESCs(T-HESCs), with final validation performed using a mouse model. Western blot and quantitative real-time polymerase chain reaction (RT-qPCR) analyses revealed that patients with adenomyosis showed a marked decrease in LEF1 expression in the stromal cells of the endometrium during the mid-secretory phase. In vitro experiments demonstrated that LEF1 knockdown in stromal cells led to impaired decidualization. Transcriptome sequencing, dual-luciferase reporter assays, and chromatin immunoprecipitation (ChIP) experiments showed that LEF1 could bind to the promoter region of interleukin (IL)-11 and promote its transcription, and IL-11 expression was also found to be downregulated in adenomyosis patients. Overexpression of IL-11 rescued the impaired decidualization caused by decreased LEF1 expression. In the in vitro co-culture model, LEF1/IL-11 knockdown led to a reduction in embryo implantation area, which was partially restored upon IL-11 overexpression. In the adenomyosis mouse model, we observed a decrease in LEF1 expression and a reduction in implantation sites compared to control mice, accompanied by impaired decidualization and receptivity. Notably, supplementation with IL-11 restored the number of implantation sites. The decrease in fertility due to reduced endometrial receptivity in adenomyosis patients is a significant clinical issue in assisted reproductive technology. This research provides insights into one potential molecular mechanism underlying this decreased receptivity, with a specific focus on the reduced expression of LEF1 in the endometrial stromal cells during the mid-secretory phase in adenomyosis patients. Our findings offer new perspectives for clinical strategies to improve endometrial receptivity in patients with adenomyosis, potentially enhancing their chances of successful pregnancy.

**Supplementary Information:**

The online version contains supplementary material available at 10.1186/s10020-024-01054-9.

## Introduction

Adenomyosis (AM) is a benign gynecological disorder characterized by invasion of the endometrial glands and stroma into the myometrium, which significantly affects women’s reproductive health (Chapron et al. 2020; Bird et al. 1972; French et al. 2022). Approximately 30% of women with AM experience fertility problems (Bourdon et al. 2020; Vercellini et al. 2023). While hormonal therapy and conservative surgery have been effective for some, numerous women still experience persistent infertility post-treatment. Research indicates that patients with adenomyosis show no significant changes in the oocyte (Horton et al. 2019), suggesting that decreased endometrial receptivity is the primary cause of infertility in AM, as confirmed by multiple studies (Munro 2019; Pirtea et al. 2021; Fischer et al. 2011).

Endometrial receptivity typically denotes the ability of the endometrium to accommodate an embryo within the period of implantation window (Kimber 2000; Ye 2020; Mahajan 2015). Endometrial decidualization refers to the transformation of endometrial stromal cells into specialized secretory cells that provide essential nutrients and an immune-privileged matrix for embryo implantation and placental development. Accordingly, any impairment in decidualization severely affects endometrial receptivity (Gellersen and Brosens 2014). Although the expression levels of homeobox A10 (HOXA10) and leukemia inhibitory factor (LIF) decrease in the mid-secretory-phase endometrium in mouse models of AM and patients with AM (He et al. 2022; Peng et al. 2021; Jiang et al. 2016), the molecular mechanisms underlying decreased decidualization and reduced endometrial receptivity in patients with AM are still not fully understood.

Lymphoid enhancer-binding factor 1 (LEF1), a member of the TCF/LEF transcription factor family, is a regulatory protein that activates or inhibits gene transcription by binding to DNA regulatory sequences and regulating transcriptional complexes. It is closely associated with cell proliferation, stem cell expansion, regeneration, and tumorigenesis and plays important roles in embryonic development and tissue homeostasis maintenance (Behrens et al. 1996; Athanasouli et al. 2023). In mice, LEF1 is involved in uterine development and gland formation and exhibits sustained high expression in the estrus but decreased expression in the metestrus and diestrus periods (Shelton et al. 2012). LEF1 is highly expressed in mouse and human endometrial cancers (Brown et al. 2023).

Interleukin (IL)-11 is a cytokine derived from stromal cells and belongs to the IL-6 family. IL-11 plays an important role in regulating endometrial receptivity, promoting decidualization, and facilitating embryo implantation. IL-11Ra deficiency leads to infertility due to impaired decidualization (Stewart et al. 1992); IL-11 expression increases significantly during the late secretory phase and early pregnancy (Cork et al. 2002; Karpovich et al. 2003).

However, the association between LEF1 and endometrial receptivity has not been reported. In this study, we investigated how the aberrant downregulation of LEF1 expression in the mid-secretory endometrium affects endometrial receptivity in patients with AM.

## Subjects and methods

### Patients

Patients diagnosed with AM through imaging, and with a history of no pregnancies after at least 3 embryo transfers (including a total of ≥ 4 good-quality embryos), were recruited for the AM group (*n* = 25). Patients with tubal obstruction or unexplained infertility who achieved a clinical pregnancy after the first embryo transfer were assigned to the control group (*n* = 25). The inclusion and exclusion criteria for all patients were as follows: the patients were 25–40 years old with regular menstrual cycles and normal endocrine profiles. Patients who used any intrauterine device or contraceptive drug within the last 6 months, or with any of the following conditions were excluded from the study: intrauterine pathology, hydrosalpinx, salpingitis, polycystic ovary syndrome, endometriosis, chromosome abnormalities, or autoimmune disease (Table 1).

**Table 1:** Clinical characteristics of women in the control and AM groups

Variable	Control (*n* = 25)	AM(*n* = 25)	*P* value
Age (y)	31.32 ± 3.35	32.56 ± 3.75	0.224
Body mass index (kg/m^2^)	21.58 ± 2.20	21.97 ± 1.51	0.469
Cycle length(days)	29.62 ± 2.06	29.74 ± 3.80	0.890
Basal FSH level (mIU/mL)	7.19 ± 1.50	7.15 ± 2.10	0.948
Basal LH level (mIU/mL)	4.65 ± 1.79	4.03 ± 1.67	0.208
Basal E_2_ level (pg/mL)	39.84 ± 16.90	39.42 ± 14.52	0.924
Basal P_4_ level (pg/mL)	0.59 ± 0.35	0.72 ± 1.07	0.537
Basal PRL level (ng/mL)	13.32 ± 5.57	14.90 ± 5.59	0.321
Basal T level (ng/mL)	0.45 ± 0.18	0.44 ± 0.17	0.830
Times of embryo transfers	1 (1,1)	5 (3,7)	< 0.0001
Average score of day 3 embryos transferred	7.75 ± 1.33	7.81 ± 1.44	0.718
Score of transferred blastocysts	3BB (3/13, 23%)	3BB (8/51, 16%)	
	4AB (6/13, 46%)	4AB (32/51, 62%)	
	4BB (4/13, 31%)	4BB (11/51, 22%)	

Data are expressed as mean ± SD or frequency. The diference between the controls and patients with AM was analyzed by independent samples t-test except “times of embryo transfers and " Average score of day 3 embryos transferred”,which were calculated by independent samples Mann-Whitney U-test (median, range). AM, adenomyosis

Endometrial specimens were collected at day LH + 7 (days 20–23 of the menstrual cycle, mid-secretory phase) using pipe suction curettage (LILYCLEANER, China). For isolation of primary endometrial cells, endometrium samples (days 11–14 of the cycle, late proliferative phase) were collected from the patients.

Samples are collected during the mid-secretory phase to study the optimal receptivity of the endometrium. Therefore, collecting cells at this stage allows for the verification of gene expression associated with these important functional alterations.

In contrast, samples are collected during the late proliferative phase (days 11–14) for primary cell culture due to practical considerations, making sample collection easier and more efficient.

For detailed information about the experimental design, see Additional file.

### Isolation of human endometrial stromal cells (HESCs) and cell cultures

Endometrial tissues were washed, minced, and digested with collagenase IV (17104019, Gibco, Waltham, MA, USA). All endometrial stroma cells were collected from the mixture after passing through a 40 μm sieve. HESCs were obtained by seeding the stromal cells on cell culture dishes, and passage 2–3 stromal cells were used to ensure the purity of the cell.

All isolated cells were cultured in DMEM/F12, 1% penicillin/streptomycin (Gibco), and 10% fetal bovine serum (Gibco), and T-HESCs were cultured in 89% DMEM/high glucose, 1% penicillin/streptomycin, and 10% fetal bovine serum in a 37 °C incubator containing 5% CO_2_.

### cDNA preparation and quantitative real-time polymerase chain reaction (RT-qPCR)

RNA was extracted from endometrial biopsy tissues, HESCs, or TERT-immortalized human endometrial stromal cells (T-HESCs) and then reverse-transcribed into cDNA according to the manufacturer’s instructions (R222, Vazyme, China). RT-qPCR was performed with the SYBR Premix Ex Taq kit (Q711, Vazyme, China) and Prism 7500. Fold change in gene expression was calculated using the 2^–ΔΔCt^ method with *GAPDH* as the reference gene. The primer pairs used in this study are listed in Additional file.

### Western blotting

Proteins from endometrial tissues and HESCs were extracted by RIPA buffer (89900, Thermo Fisher Scientific, Waltham, MA, USA) containing a protease inhibitor cocktail (5892970001, Roche, Switzerland). Proteins were electroblotted onto 0.22 μm polyvinylidene difluoride membranes (ISEQ00010, Millipore, Burlington, MA, USA) after being separated by 10% and 12.5% SDS-PAGE (PG112, PG113, Epizyme, China). Membranes were successively incubated with primary and secondary antibodies (listed in Additional file), followed by blocking with 3% skim milk. Enhanced chemiluminescence analysis (E412-01, Vazyme, China) was performed to obtain the images.

### Immunohistochemical staining (IHC)

The tissues were preserved using 4% paraformaldehyde (BL539A, Biosharp, China) and then embedded in paraffin blocks. Each 5 μm-thick section underwent deparaffinization, rehydration, antigen retrieval, and a 10-min submersion in 3% hydrogen peroxide. The sections were then washed with Tris-buffered saline and blocked using 5% normal goat serum for 30 min. Following this, they were incubated with either anti-LEF1, anti-IL-11, anti-HAND2, anti-LIF, or rabbit /rat monoclonal IgG isotype control at 4 °C for 12 h. Sections were then exposed to secondary antibodies, stained with diaminobenzidine, and counterstained with hematoxylin. Visualization was achieved using a Nikon Eclipse 80i microscope (Japan). The intensity of immunostaining was evaluated using the following H-Score (Zhou et al. 2020):

$$\eqalign{& \:H - Score\:(\sum \: (pi \times \:i) \cr & = (percentage\:of\:weak\:intensity\:cells \times \:1) \cr & + (percentage\:of\:moderate\:intensity\:cells \times \:2) \cr & + (percentage\:of\:strong\:intensity\:cells \times \:3) \cr}$$

Each section was scored by two independent pathologists, and the results were then analyzed.

### Enzyme-linked immunosorbent assay (ELISA) analysis

IL-11, insulin-like growth factor-binding protein 1 (IGFBP1), and prolactin (PRL) expression levels were measured using ELISA kits (EK111, EK1255, and EK1304, Multisciences, China). The process involved adding 50 µl of culture medium to the samples, as per the instructions provided by the manufacturer. After the addition of the samples and the creation of standard curves, the optical density was measured at a wavelength of 450 nm using a Multiskan GO instrument.

For endometrial specimens, 50–100 mg of tissue samples were weighed. Five times the mass volume of 1X PBS buffer containing a 1% protease inhibitor cocktail (5892970001, Roche) was added to the samples. Scissors or similar tools were used to finely chop the tissue samples. The samples were subjected to ultrasonic cell disruption while being kept on ice. Following this, they were incubated at 4 °C or maintained on ice for a duration of 30 min. Thereafter, the samples were centrifuged for 10 min at 12,000 × g, and the supernatant was collected. The levels for each sample were normalized to the amount of the total protein.

### Immunofluorescence

Slices of human endometrial tissues and cultured cells were fixed with 4% PFA, permeabilized with 0.3% Triton X-100, blocked with 5% bovine serum albumin, and incubated with primary antibodies. The sections were incubated with secondary antibodies for 1 h, followed by Hoechst 33342 staining for 15 min at room temperature. Images were captured using a fluorescence microscope (Zeiss-Axio Vert.A1, Germany; Leica Thunder DMi8, Germany) or a confocal laser scanning microscope (Leica, sp8). Information on the antibodies used is provided in Additional file.

For cytoskeleton staining, TRITC-phalloidin (G1041, Servicebio, China) was used in accordance with the manufacturer’s guidelines.

### Separation and preparation of cytoplasmic and nuclear extracts of HESCs

Proteins from the cytoplasm and nucleus of HESCs were extracted using a nuclear and cytoplasmic protein extraction kit (78833, Thermo Fisher Scientific) following the manufacturer’s instructions. GAPDH and LAMIN-B1 were used as markers for the cytoplasmic and nuclear fractions, respectively.

### Lentiviral inhibition and plasmid overexpression in cells

Cells were transduced with either scramble shRNA, *LEF1*-shRNA, or *IL-11*-shRNA (2 × 10^7^ transducing units/mL) using polybrene (10 µg/ml). Following transduction, the cells underwent selection with G418 (1000 µg/ml) and were subsequently cultured in the presence of G418 reagent (500 µg/ml). The lentiviruses were purchased from Hanbio (China). The scramble and target sequences of gene interference are provided in Additional file. *IL-11* and negative plasmids purchased from GeneCopoeia (Rockville, MD, USA) were transfected into cells using X-tremeGENE HD DNA transfection reagent (06366236001, Roche), according to the manufacturer’s instructions.

### In vitro decidualization assay

HESCs and T-HESCs were cultured in serum-free, phenol red-free DMEM/F12 (LD1230, Bioagrio, China) supplemented with medroxyprogesterone acetate (MPA, 1 µM, HY-B0469, MCE) and 8-bromoadenosine 3’,5’-cyclic monophosphate (8-Br-cAMP, 1 mM, ab141448, Abcam, USA) for 3 and 5 days, respectively. Markers of decidualization (PRL and IGFBP1) were analyzed using RT-qPCR, ELISA, and western blotting.

### Embryo outgrowth analysis

HESCs were harvested from the endometrium during the late proliferative phase and then seeded in a 24-well plate. After a period of 72 h, these cells once transfected with scramble shRNA, *LEF1*-shRNA, IL-11-shRNA, negative plasmids, or *IL-11* overexpression plasmids underwent the decidualization process as previously described. Mouse blastocysts, which exhibited a standard morphology and had successfully hatched, were co-cultured alongside the confluent monolayers of the decidualized HESCs using a DMEM/F12 complete medium. The areas of trophoblast outgrowth were meticulously marked, and their measurements were computed using the ImageJ software (NIH, Bethesda, MD, USA).

### Whole-genome expression profile and bioinformatics analysis

T-HESCs transfected with scramble shRNA or *LEF1*-shRNA for three days were collected using the Trizol reagent (Thermo Fisher Scientific). The sequencing library was then sequenced on the NovaSeq6000 platform (Illumina) with the assistance of Genekinder Medicaltech (Genekinder Medicaltech Co., Ltd., China). Paired-end sequence files (fastq) were mapped to the reference genome using Hisat2 (v2.0.5). The TPM algorithm was used for normalization. Differential expression analysis for mRNA was performed using the R package DESeq2. An illustration of differentially expressed genes (DEGs) between the two samples was shown in volcano plots generated by the R package (v4.1.0) on the Hiplot Platform (https://hiplot.com.cn).

### Dual-luciferase reporter gene assay

The interaction between *LEF1* and the *IL-11* promoter region was explored using a dual-luciferase reporter assay. Both wild-type *IL-11* promoter luciferase and its mutant counterpart were synthesized by Genomeditech Co., Ltd. (China). HEK293T cells were cultured in 24-well plates. For each well, 300 ng of luciferase reporter constructs were co-transfected with 600 ng of either pcDNA-NC or pcDNA- *LEF1* using Lipofectamine 2000 (Invitrogen, Waltham, MA, USA). After an incubation period of 48 h, the Dual-Luciferase Reporter Assay kit, sourced from Genomeditech, China, was employed to gauge the relative luciferase activity.

### Chromatin immunoprecipitation (ChIP) assay

ChIP was conducted using T-HESCs. Cells were cross-linked by adding 37% formaldehyde to a final concentration of 1% for 10 min at room temperature. The subsequent steps followed the manufacturer’s instructions for the ChIP assay kit (17-10086, Merck, Rahway, NJ, USA). The enriched DNA was then analyzed using specific primers via RT-qPCR.

### Animal models and methods for adenomyosis induction

In our study, we induced adenomyosis in female neonatal CD-1 mice (AM-model group) through oral administration of 1 mg/kg tamoxifen (HY-13757 A, mce), dissolved in a mixture of peanut oil, lecithin, and condensed milk (in a 2:0.2:3 volume ratio), with a dosage of 5 µl/g of body weight, from the first to the fifth day post-birth (Bourdon et al. 2023). Meanwhile, control female neonatal CD-1 mice (vehicle group) received an equivalent volume of the solvent mixture without tamoxifen. Upon reaching 21 days of age, all mice were weaned and separated from their mothers.

All CD-1 mice purchased from Vital River Laboratory Animal Technologies Co. Ltd. were housed in SPF with regular diet and diurnal rhythm.

### Evaluation of pregnancy outcomes

Female mice at 10 weeks of age were mated with fertile wild-type males, with the detection of a vaginal plug marking day 1 of pregnancy. The day before mating, TAM treatment mice received IL-11 (200 mg/kg) intravenously, whereas control mice were injected with a comparable volume of saline solution. Females showing evidence of mating (plug-positive) were isolated for pregnancy-related studies. On the seventh day of pregnancy, implantation sites in these mice were identified through intravenous administration of 200 µL of 1% Evans Blue in saline, with the sites indicated by clear blue bands. For each experimental condition in every mouse model, a minimum of three mice were used.

### Statistical analysis

For normally distributed data with homogeneity of variance, Student’s t-test was used to compare data between two groups, and one-way analysis of variance (ANOVA) with Bonferroni’s test or Dunnett’s test was used to compare data between more than two groups. For nonparametric data, appropriate nonparametric tests were applied. All statistical analyses were performed using SPSS software (version 22.0; IBM Corp., Armonk, NY, USA) and GraphPad Prism software (version 6.0; GraphPad Inc., La Jolla CA, USA). All experiments were conducted at least three times, and statistical significance was defined as *P* < 0.05.

## Results

### Patients with AM exhibited insufficient endometrial decidualization and reduced endometrial receptivity

**Fig. 1:**
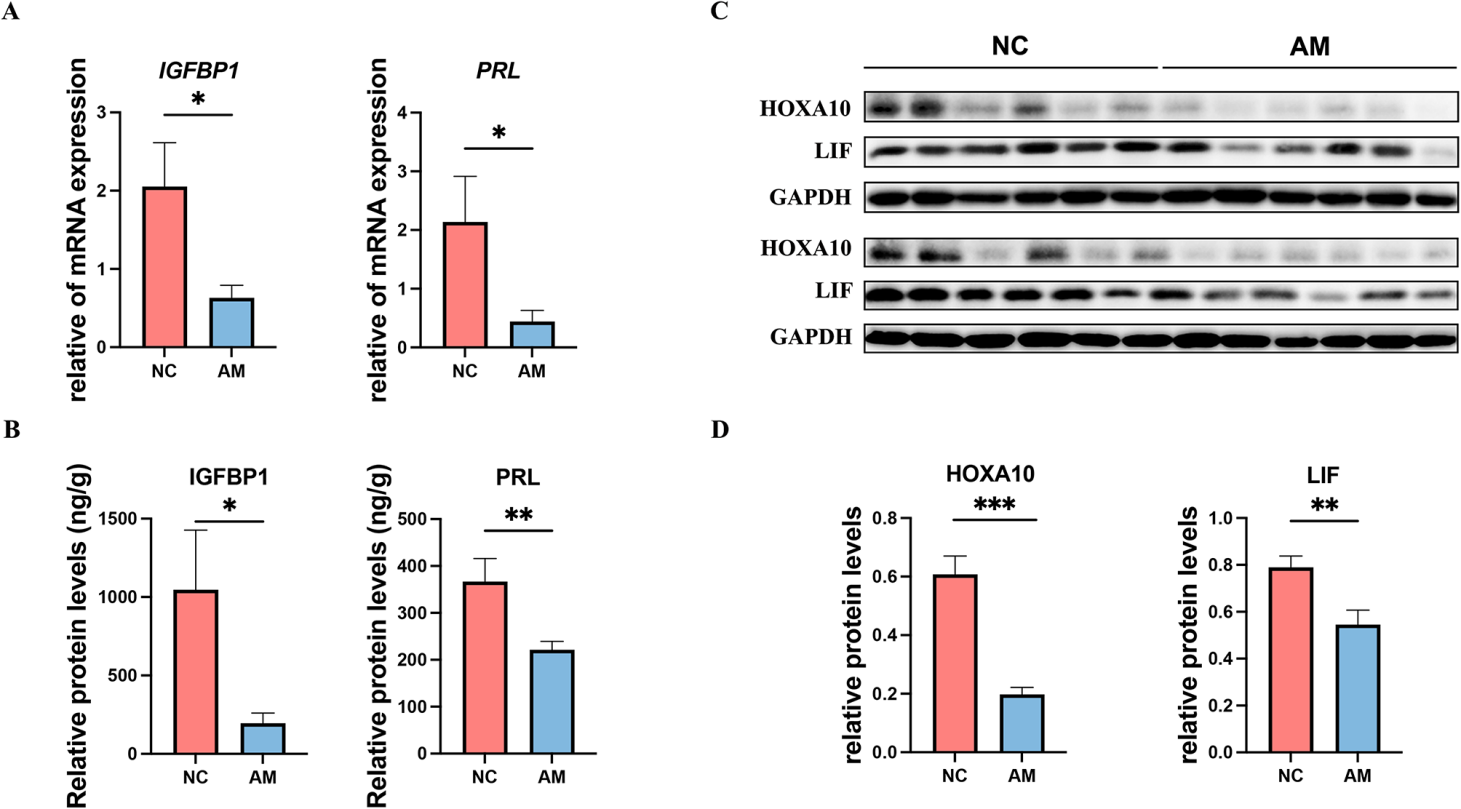
Endometrial decidualization and receptivity levels in patients with adenomyosis. The mRNA and protein levels of IGFBP1 and PRL in the endometrium in the mid-secretory endometrium were measured by RT-qPCR (**A**, *n* = 50) and ELISA (**B**, *n* = 50). The protein levels of HOXA10 and LIF in the mid-secretory endometrium of control and patients with adenomyosis were measured using western blotting (**C**–**D**, *n* = 24). Statistical analysis was performed using Student’s t-test. Data are presented as mean ± SEM. *P < 0.05; **P < 0.01; ***P < 0.001. The data were considered statistically significant at *P* < 0.05. AM, adenomyosis group; NC, negative control group; SEM, standard error of the mean

We collected mid-secretory endometrium samples from patients with AM and a control group and examined the expression of markers for decidualization and endometrial receptivity. RT-qPCR and ELISA results demonstrated that mRNA (Fig. 1A) and protein (Fig. 1B) levels of the decidualization markers IGFBP1 (*p* = 0.010/0.030) and PRL (*p* = 0.042/0.003) were decreased in patients with AM. Western blotting results demonstrated that the endometrial receptivity markers HOXA10 (*p*<0.001) and LIF (*p* = 0.005) had reduced expression levels in patients with AM compared with those in the control group (Fig. 1C and D). These findings suggest that insufficient decidualization in patients with AM may be an important contributing factor to their reduced endometrial receptivity.

### LEF1 was decreased in the endometrial stroma of patients with AM compared with that in controls

**Fig. 2:**
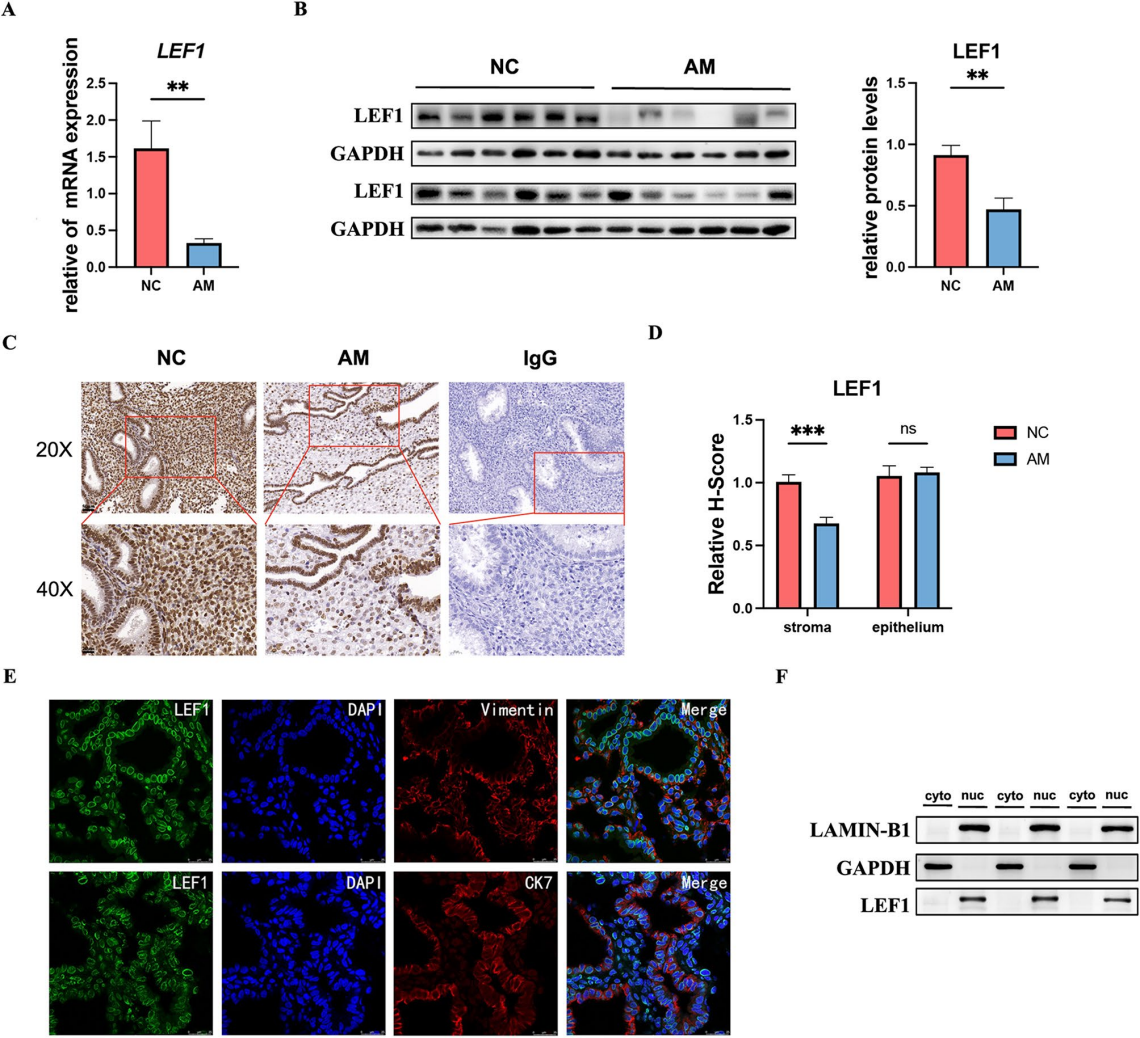
LEF1 expression in the human endometrium. The mRNA and protein levels of LEF1 in the endometrium were measured using RT-qPCR (**A**, *n* = 50). Western blotting (**B**,*n* = 24) and immunohistochemistry (**C** and **D**, *n* = 20, share scale bars, 50 and 20 μm). Co-localization of LEF1 (green fluorescence) with stromal cell markers (vimentin and red fluorescence) and epithelial cell markers (CK7 and red fluorescence) (**E**, *n* = 10). Western blot analysis of nuclear and cytoplasmic proteins in HESCs (**F**, *n* = 3). Statistical analysis was performed using Student’s t-test. Data are presented as mean ± SEM. *P < 0.05; **P < 0.01. Data were considered statistically significant at *P* < 0.05. CK7, cytokeratin; DAPI, nuclear staining. AM, adenomyosis group; NC, negative control; SEM, standard error of the mean

LEF1 expression in the mid-secretory endometrium of patients with AM revealed a decrease in both LEF1 mRNA and protein levels, as indicated by RT-qPCR (Fig. 2A, *p* = 0.002), western blotting (Fig. 2B, *p* = 0.002), and IHC (Fig. 2C). For analyzing the IHC data, we separately assessed the expression of LEF1 in epithelial and stromal cells, revealing significant differences primarily in stromal cells (Fig. 2D, *p* = 0.012). To further investigate its cellular localization, we performed immunofluorescence co-localization and nuclear-cytoplasmic fractionation experiments targeting HESCs (Fig. 2E and F), which confirmed that LEF1 was predominantly expressed in the cell nucleus.

### Knocking down LEF1 decreased decidualization in T-HESCs and HESCs

**Fig. 3:**
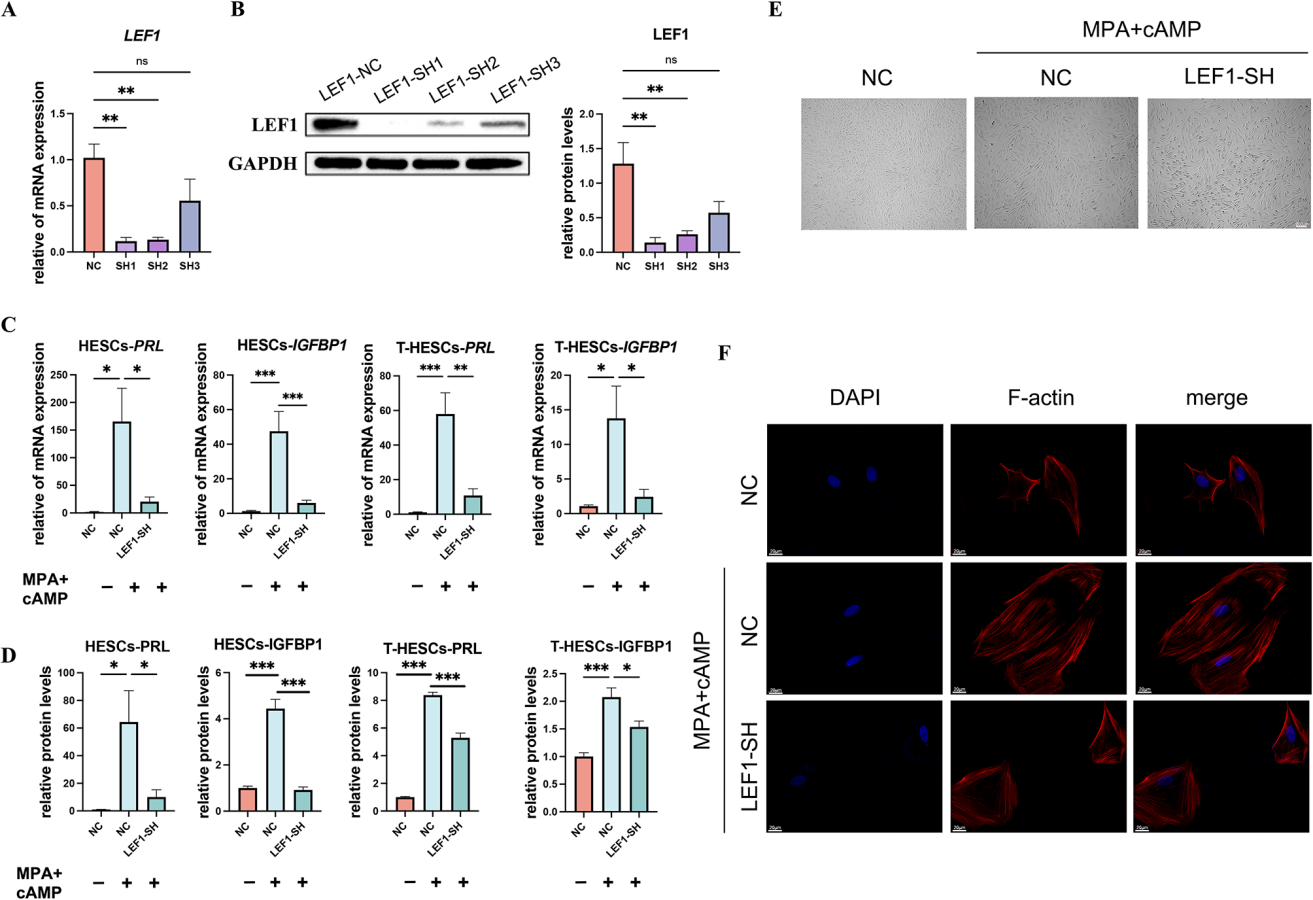
Changes in cell decidualization following LEF1 knockdown. The LEF1 knockdown efficiency was determined using RT-qPCR (**A**) and western blot analysis (**B**). The mRNA and protein levels of PRL and IGFBP1 were measured using RT-qPCR (**C**) and ELISA (**D**) using T-HESCs and HESCs. The cell morphology of HESCs transfected with scramble shRNA or LEF1-sh was examined under light microscopy (**E**, share scale bars, 200 μm) and visualized using TRITC-phalloidin (**F**, share scale bars, 20 μm) after a 3-day decidualization treatment. Significance was determined using one-way ANOVA with Dunnett’s test (**A** and **B**) and Bonferroni’s test (**C** and **D**), respectively. Data are presented as mean ± SEM.*P < 0.05; **P < 0.01; ***P < 0.001. Data were considered statistically significant at *P* < 0.05. SEM, standard error of the mean; ANOVA, analysis of variance

The effects of LEF1 knockdown in T-HESCs were examined using RT-qPCR and western blotting (Fig. 3A and B). To investigate the involvement of LEF1 in decidualization, we established an in vitro model using T-HESCs and HESCs, in which the cells were treated with MPA and 8-bromo-cAMP to induce decidualization. Our findings demonstrated that LEF1 knockdown in both T-HESCs and HESCs decreased the mRNA (Fig. 3C) and protein (Fig. 3D) expression levels of the decidualization markers PRL and IGFBP1. Considering the distinct morphological differences between decidualized and non-induced cells, we observed these discrepancies under light and fluorescent microscopes. Specifically, induced decidualized cells exhibited an enlarged and rounded morphology, whereas LEF1 knockdown resulted in the loss of these characteristic features (Fig. 3E and F). This further underscores the detrimental effects of reduced LEF1 expression on decidualization.

### LEF1 enhanced the transcriptional activity of IL-11

**Fig. 4:**
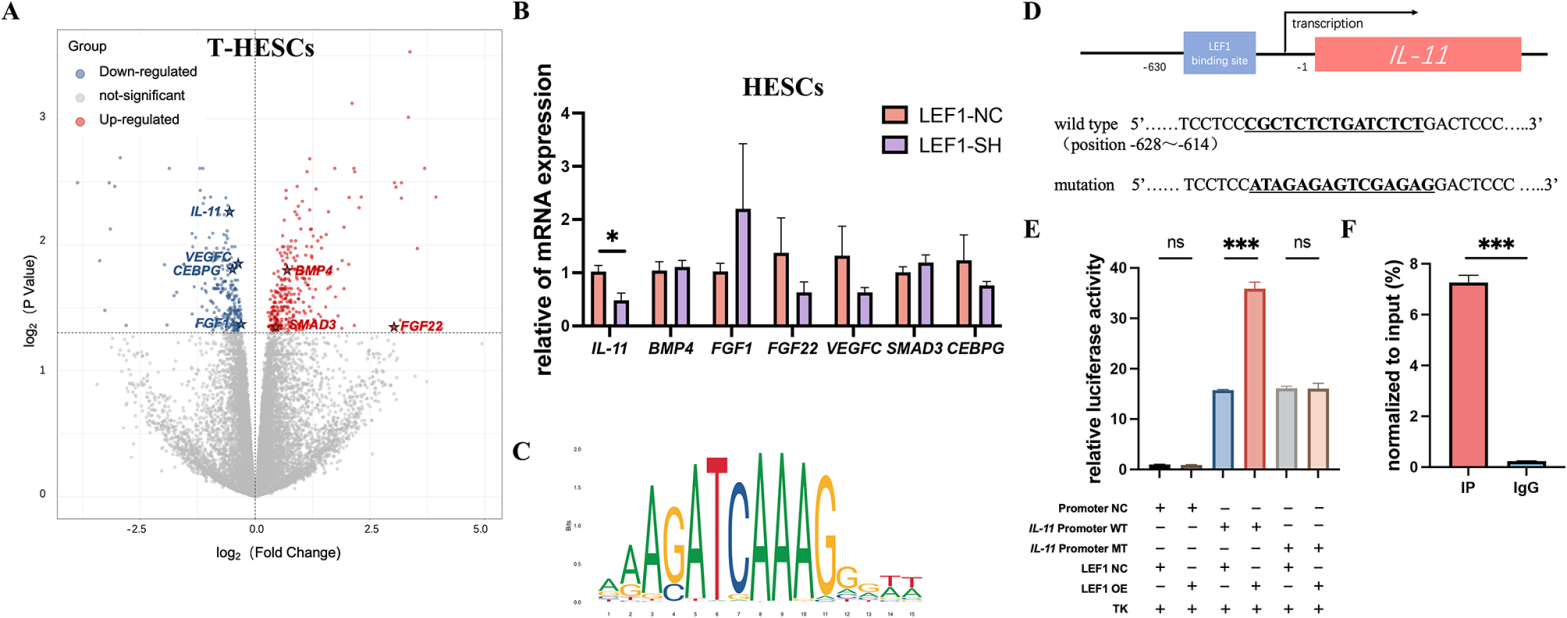
LEF1 regulates IL-11 expression. The volcano plot illustrates the 563 genes that were statistically significant (*P* < 0.05) (**A**, *n* = 8). Differentially expressed genes involved in endometrial receptivity and implantation were validated by RT-qPCR (**B**). Motif of LEF1 predicted on the JASPAR system(**C**). The constructs of the human IL-11 promoter included both wild-type (WT) LEF1 binding elements (CBEs) and mutated CBEs, with the mutated sequences underlined (**D**). The IL-11 promoter activity was evaluated using a dual-luciferase assay (**E**). Chromatin immunoprecipitation (ChIP) analysis of LEF1 binding to the IL-11 promoter (**F**). Significance was determined using one-way ANOVA with Bonferroni’s test (**B** and **E**) and Student’s t-test (**F**), respectively. Data are presented as mean ± SEM.*P < 0.05; ***P < 0.001. Data were considered statistically significant at *P* < 0.05. SEM, standard error of the mean; ANOVA, analysis of variance

To further investigate the molecular mechanisms underlying impaired decidualization and reduced endometrial receptivity in patients with AM due to decreased LEF1 expression, we performed RNA sequencing analysis of LEF1-SH of T-HESCs and conducted an extensive literature review. Among the DEGs identified from the sequencing data, seven were associated with decidualization (Fig. 4A). Upon validation using HESCs, we noted that only IL-11 exhibited statistically significant and consistent differential expression in accordance with the sequencing results (Fig. 4B, *p* = 0.025). Using the JASPAR website, we conducted a predictive analysis and identified one putative binding site for LEF1 as a transcription factor within the *IL-11* promoter region (Fig. 4C and D). We conducted dual-luciferase reporter assays with site mutations to probe the interaction and chose predicted sequences for chromatin immunoprecipitation (ChIP) assays. (Figs. 4E and F). Our results revealed that overexpression of LEF1 significantly enhanced the fluorescence intensity in the wild-type promoter region, providing further evidence that LEF1 can modulate the transcriptional activity of *IL-11*.

### IL-11 expression was decreased in the mid-secretory phase of the endometrium in patients with AM

**Fig. 5:**
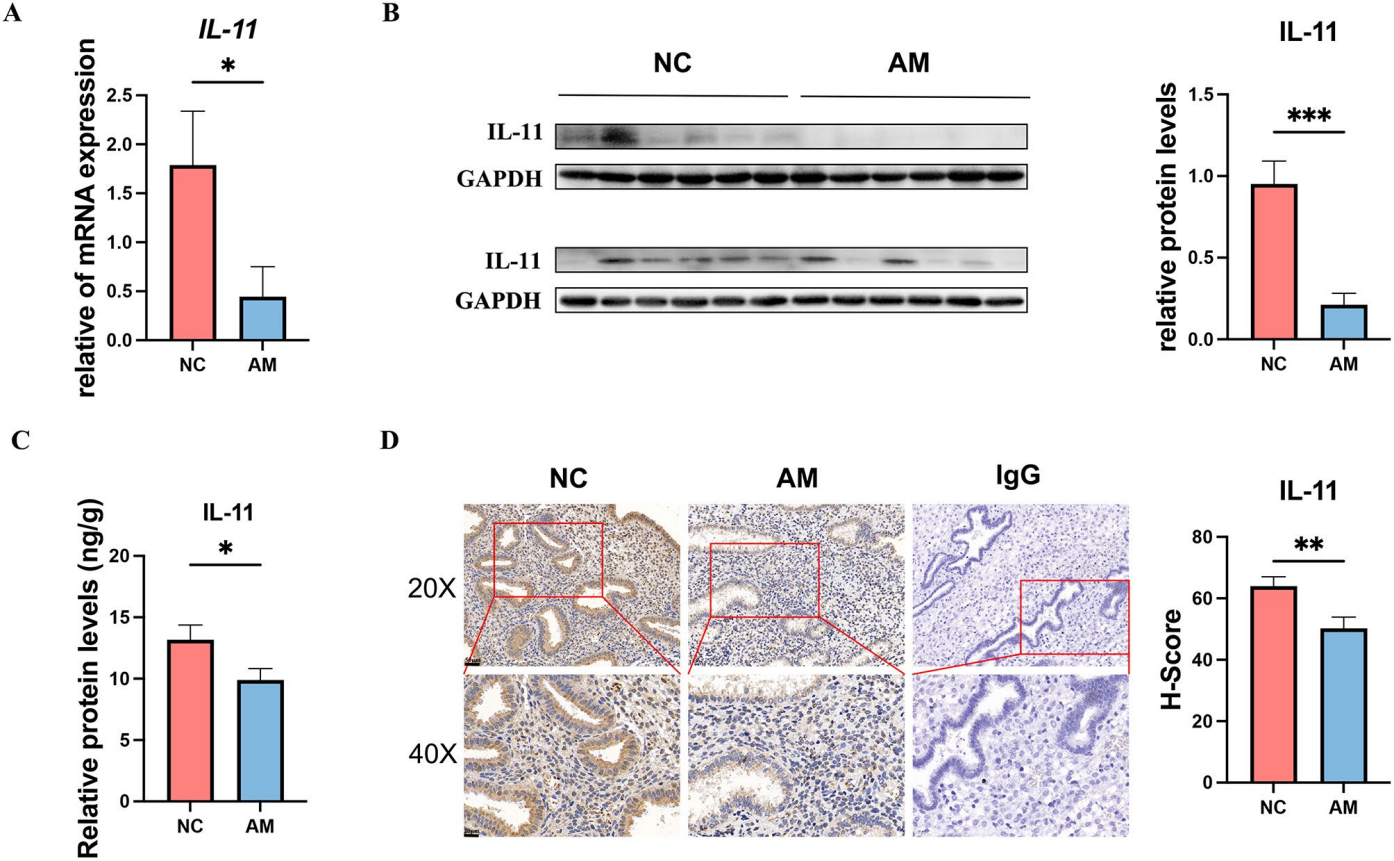
The expression of IL-11 was decreased in the endometrium of patients with adenomyosis. The mRNA expression levels of IL-11 were measured using RT-qPCR (**A**, *n* = 50). The protein expression levels of IL-11 were measured using western blotting (**B**, *n* = 24), ELISA (**C**, *n* = 50), and IHC (**D**, *n* = 20, 20X scale bars, 50 μm; 40X scale bars, 20 μm). Statistical analysis was performed using Student’s t-test. Data are presented as mean ± SEM. * *P* < 0.05. Data were considered statistically significant at *P* < 0.05. AM, adenomyosis group; NC, negative control; SEM, standard error of the mean

We investigated the expression levels of IL-11 mRNA and protein in endometrial tissues obtained from both the control and AM groups in the mid-secretory phase of the endometrium. Our findings demonstrated a significant downregulation of IL-11 expression in the endometrium of the AM group compared with that in the control group (Fig. 5A, *p* = 0.044;B, *p*<0.001; C, *p* = 0.037,D).

### Knockdown of IL-11 reduced decidualization in T-HESCs and HESCs

**Fig. 6:**
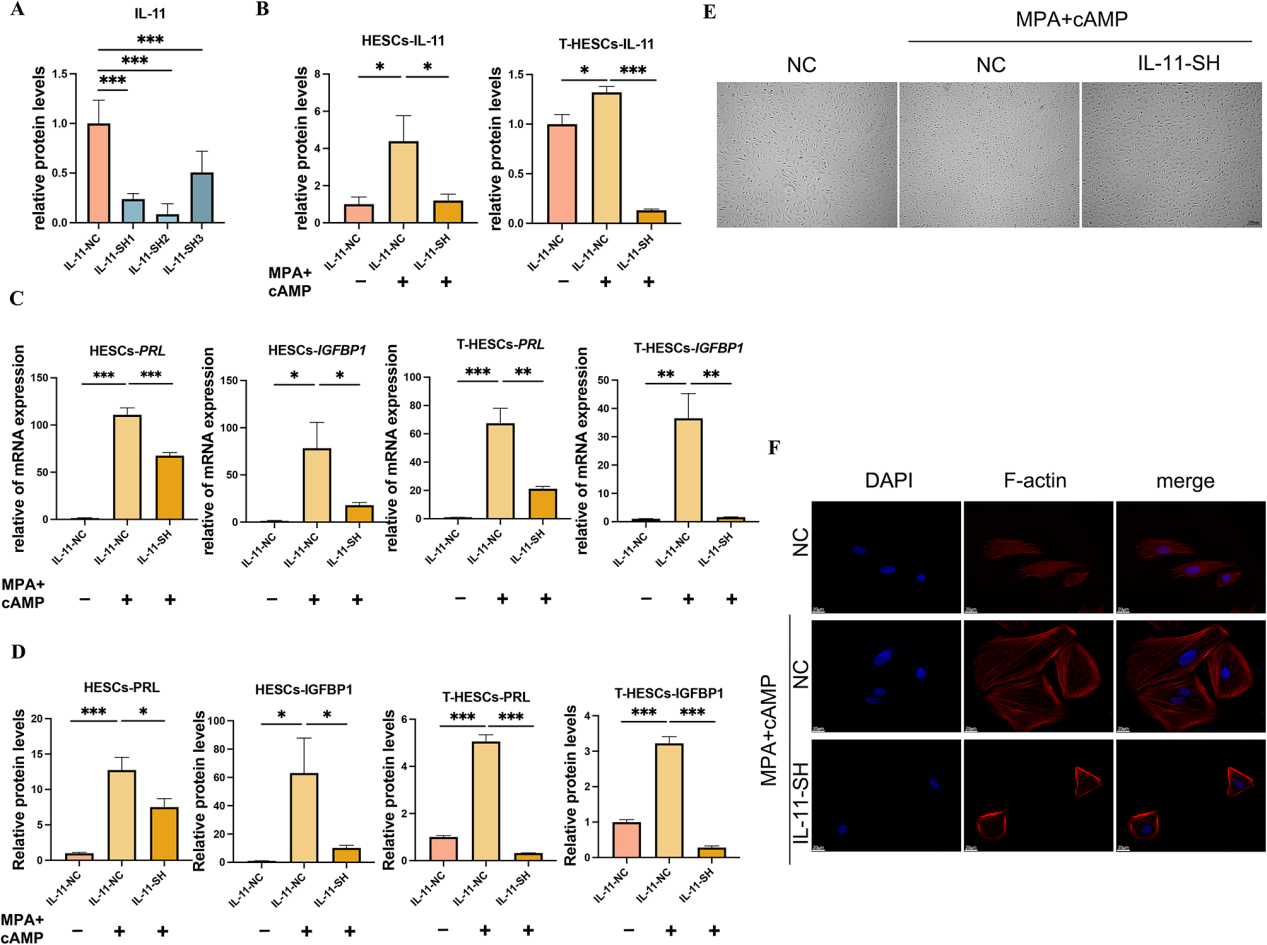
Changes in cell decidualization following IL-11 knockdown. The IL-11-knockdown efficiency was shown by ELISA (**A**) in T-HESCs. The protein levels of IL-11 were measured using ELISA (**B**) in HESCs and T-HESCs. The mRNA and protein levels of PRL and IGFBP1 were measured using RT-qPCR (**C**) and ELISA (**D**), respectively, in T-HESCs and HESCs. The cell morphology of HESCs was observed under light microscopy (**E**; scale bars, 200 μm) and visualized using TRITC-phalloidin (**F**; scale bars, 20 μm.) after a 3-day decidualization treatment. Significance was determined using one-way ANOVA with Dunnett’s test (**A**) and Bonferroni’s test (**B**-**D**), respectively. Data are presented as mean ± SEM.*P < 0.05; **P < 0.01; ***P < 0.001. Data were considered statistically significant at *P* < 0.05. SEM, standard error of the mean; ANOVA, analysis of variance

To investigate the functionality of IL-11 further, we constructed relevant lentiviral vectors and selected the sequence with the highest knockdown efficiency for subsequent experiments (Fig. 6A). The results revealed that IL-11 expression significantly increased after decidualization induction. However, the knockdown of IL-11 in stromal cells resulted in decreased expression levels of IGFBP1 and PRL, as well as the loss of characteristic morphological changes associated with decidualization (Fig. 6B–F). This indicates that IL-11, like LEF1, influences decidualization.

### IL-11 overexpression rescued impaired decidualization caused by reduced LEF1

**Fig. 7:**
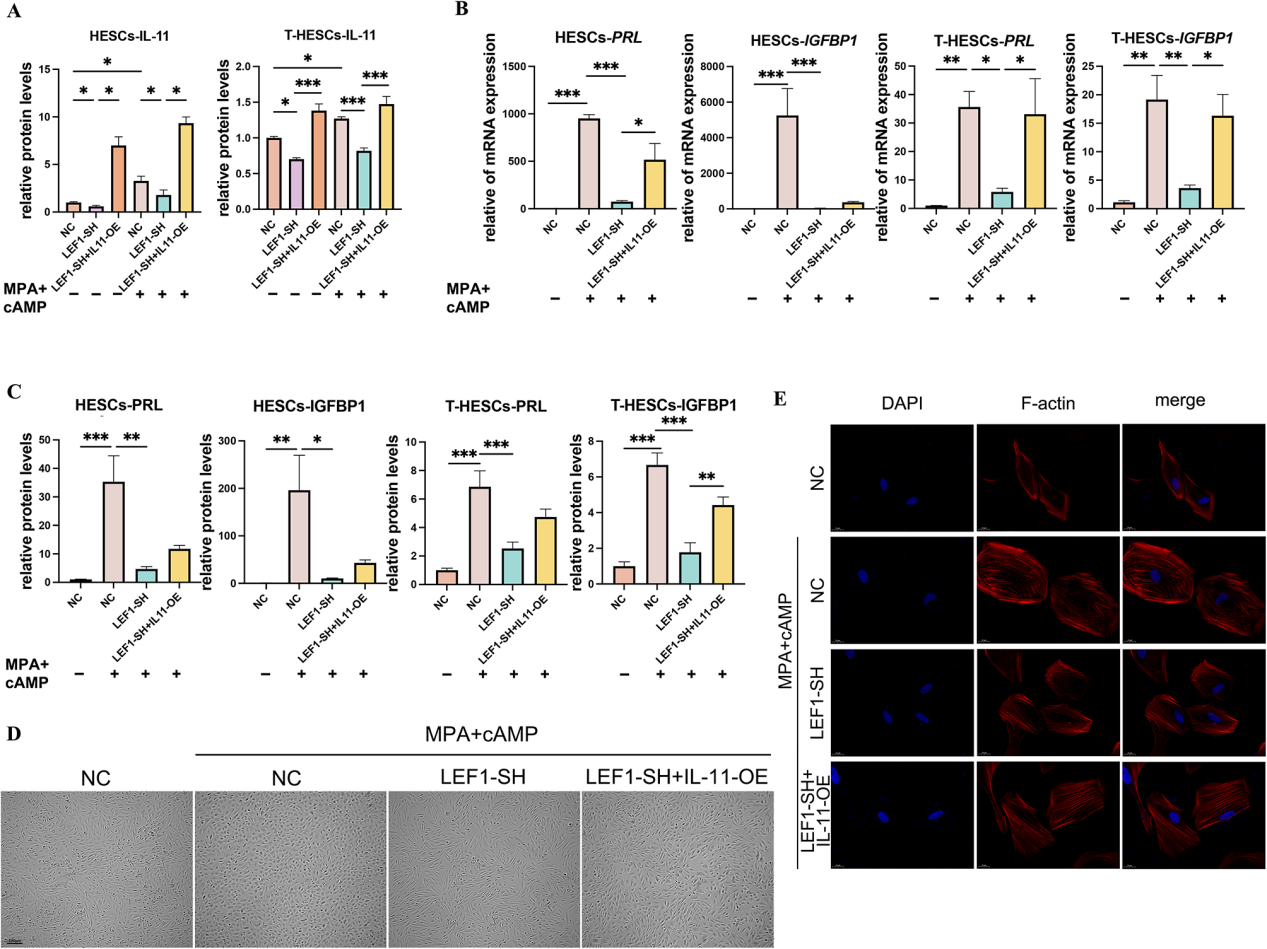
Impact of IL-11 overexpression on decidualization changes in the context of reduced LEF1 expression. Protein levels of IL-11 were measured by ELISA (**A**). The mRNA and protein levels of PRL and IGFBP1 were measured using RT-qPCR (**B**) and ELISA (**C**), respectively, in T-HESCs and HESCs. The cell morphology of HESCs was examined under light microscopy (**D**; scale bars, 200 μm) and visualized using TRITC-phalloidin (**E**; scale bars, 20 μm) after a 3-day decidualization treatment. Significance was determined using one-way ANOVA with Bonferroni’s test. Data are presented as mean ± SEM.*P < 0.05; **P < 0.01; ***P < 0.001. Data were considered statistically significant at *P* < 0.05. SEM, standard error of the mean; ANOVA, analysis of variance

To examine whether LEF1 affects decidualization by regulating IL-11, we performed rescue experiments by overexpressing IL-11 based on LEF1 knockdown. LEF1 knockdown resulted in decreased IL-11 expression. Furthermore, the induction of decidualization promoted IL-11 expression (Fig. 7A). Similarly, we tested for IGFBP1 and PRL and noted that the decreased decidualization level caused by LEF1 knockdown could be rescued by overexpressing IL-11 (Fig. 7B–E). This indicates that LEF1 influences decidualization by regulating IL-11.

### LEF1/IL-11 knockdown in HESCs inhibited trophoblastic outgrowth in vitro

**Fig. 8:**
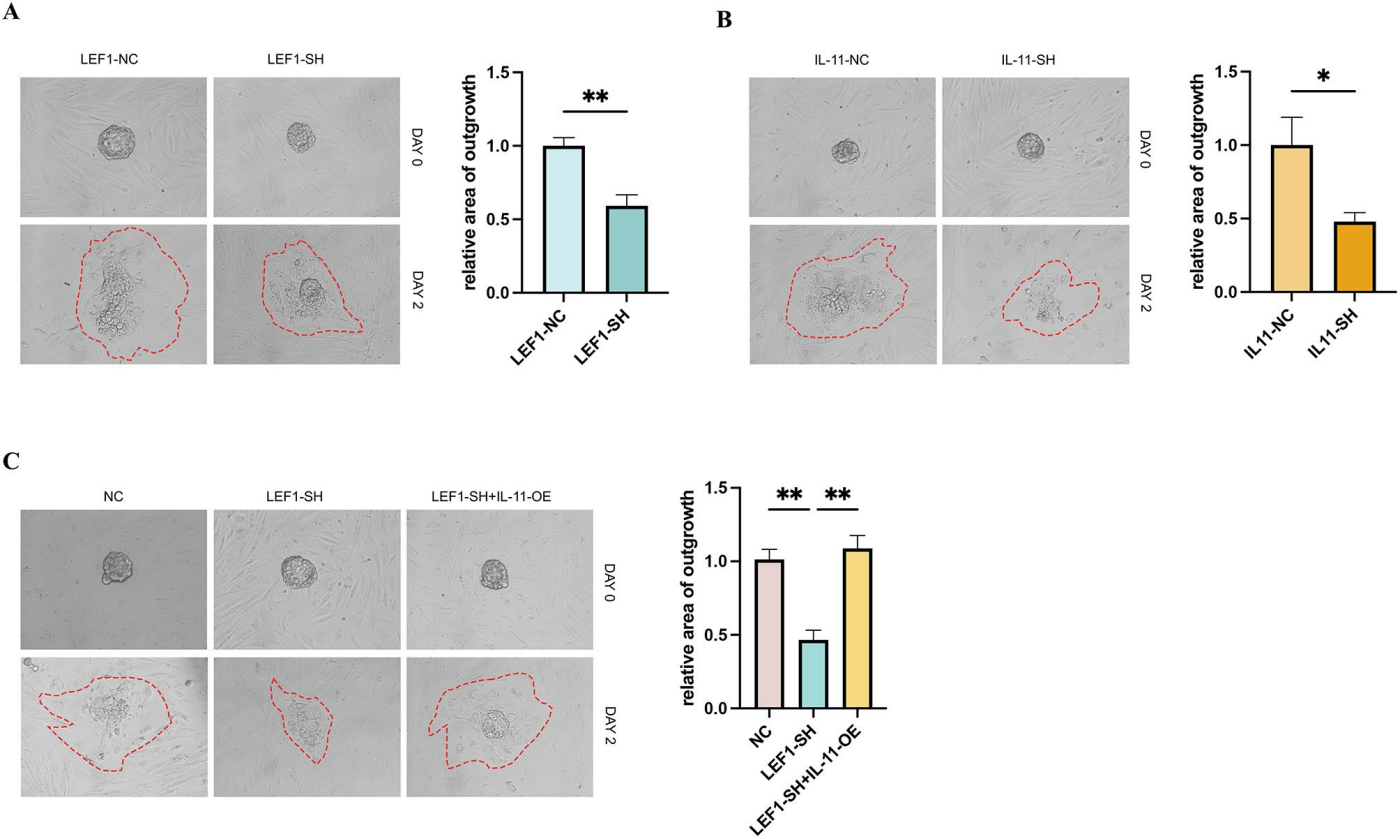
Trophoblast outgrowth in vitro assay. Representative images showing the spread of mouse trophoblasts (outlined with dotted lines) co-cultured with HESCs (**A**–**C**). The quantification of the outgrowth area was conducted with three independent samples. The average outgrowth area of the control group was normalized to 100%. Significance was determined using Student’s t-test (**A** and **B**) and one-way ANOVA with the Bonferroni’s test (**C**), respectively. Data are presented as mean ± SEM. *P < 0.05; **P < 0.01. Data were considered statistically significant at *P* < 0.05. SEM, standard error of the mean; ANOVA, analysis of variance

We employed a heterologous co-culture system to simulate trophoblast invasion into the endometrial stroma, which resembles an in vivo implantation process. After 48 h of co-culture, LEF1/IL-11-knockdown HESCs exhibited significant impairment in trophoblast outgrowth (Fig. 8A and B). However, this impairment in LEF1 expression was effectively rescued by IL-11 overexpression (Fig. 8C). This observation suggests that LEF1/IL-11 plays a crucial role in promoting supportive interactions between HESCs and trophoblasts during the early stages of embryo implantation.

### IL-11 treatment partially rescues impaired fertility in AM-model mice

**Fig. 9:**
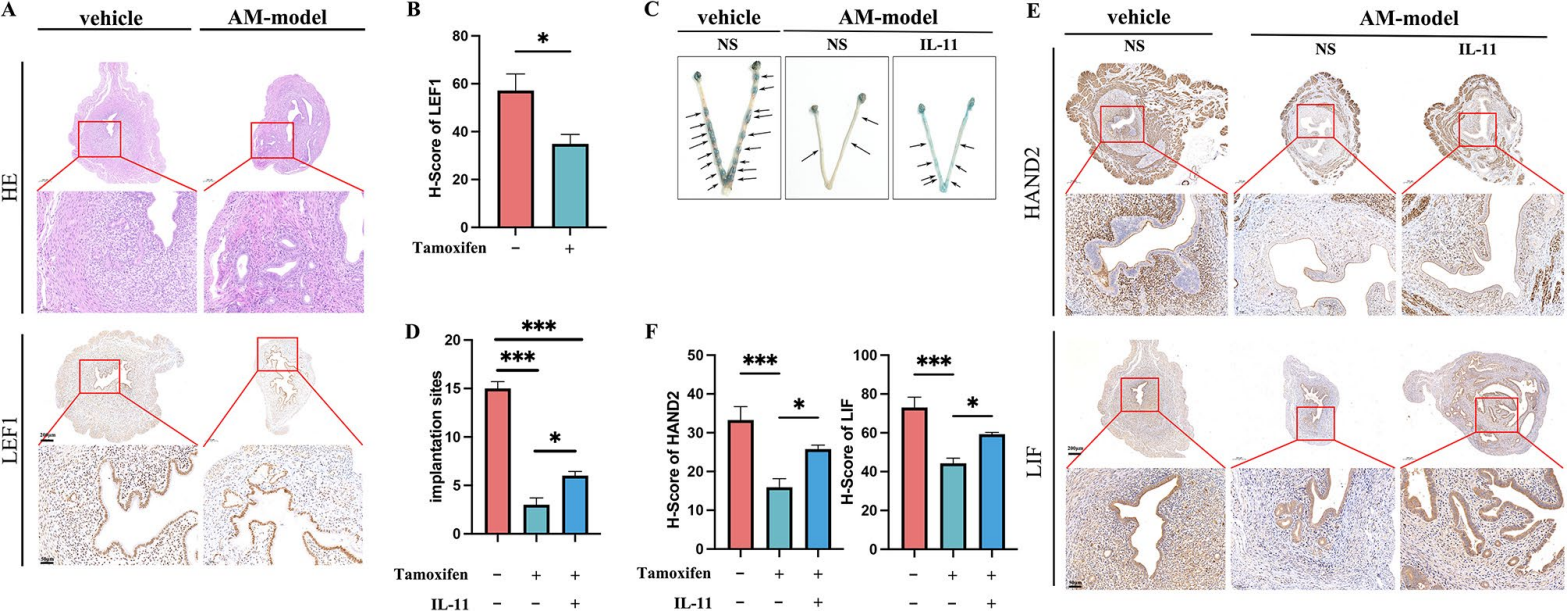
The changes in the AM-model mice. The results of Hematoxylin and eosin (HE) staining and IHC experiments of LEF1 (**A** and **B**, *n* = 10, share scale bars, 200 and 50 μm). Implantation site outcomes in mice (**C** and **D**, *n* = 30). The levels of HAND2 and LIF in the uterine of CD-1 mice were analyzed by immunohistochemistry (**E** and **F**, *n* = 30, share scale bars, 200 and 50 μm). Significance was determined using Student’s t-test (**B** and **D**) and one-way ANOVA with Bonferroni’s test (**F**), respectively. Data are presented as mean ± SEM. *P < 0.05; **P < 0.01; ***P < 0.001. Data were considered statistically significant at *P* < 0.05. SEM, standard error of the mean; ANOVA, analysis of variance; LIF, leukemia inhibitory factor

In tamoxifen-induced mice, the observed disruption of endometrial structure and invasion of endometrial glands and stroma into the myometrium, reflecting the pathological features of adenomyosis, indicate the successful construction of AM model. Simultaneously, the decreased expression of LEF1 in the endometrial stromal cells of adenomyosis mice aligns with the findings observed in patients with adenomyosis (Fig. 9A and B). The implantation sites in adenomyosis model mice significantly decreased compared with those in vehicle mice (Fig. 9C and D). Subsequent analysis revealed a distinct decrease in the expression of decidualization marker HAND2 and endometrial receptivity marker LIF in the adenomyosis model (Fig. 9E and F). Notably, administration of IL-11 partially ameliorated these observed alterations (Fig. 9C-F).

## Discussion

Increasing evidence suggests that AM adversely affects female fertility, particularly during assisted reproduction (Vercellini et al. 2023; Horton et al. 2019). The primary factor contributing to infertility in AM is reduced endometrial receptivity, which hinders a successful pregnancy (Munro 2019). Impaired decidualization is considered to be the main factor contributing to reduced endometrial receptivity in individuals with AM (Mahajan 2015; Gellersen and Brosens 2014; He et al. 2022); however, the specific molecular mechanisms remain unclear. Our study revealed that decreased expression of LEF1/IL-11 during the mid-secretory phase inhibits decidualization of human endometrial stromal cells, which may contribute to reduced fertility in patients with AM.

Our study revealed that decreased expression of LEF1/IL-11 during the mid-secretory phase inhibits decidualization of human endometrial stromal cells, which may contribute to reduced fertility in patients with AM.

LEF1, a high-mobility group transcription factor, belongs to the TCF/LEF transcription factor family within the WNT signaling pathway. The role of the WNT signaling pathway is crucial in uterine endometrial decidualization and embryo implantation (Sonderegger et al. 2010; Dietrich et al. 2022; Zhang and Yan 2015). LEF1 plays a vital role in cell growth and organ development, and the aberrant expression of LEF1 may disrupt normal cell regulatory mechanisms (Eliason et al. 2020; McGraw et al. 2011). Although it has been extensively studied in the context of cancer and immunology, being considered crucial for early T cell development (Heino et al. 2021; Kaer 2023; Ngai et al. 2023), research into LEF1 in reproductive medicine, especially regarding endometrial receptivity, has been comparatively scarce. Here, we observed a significant decrease in the expression of LEF1 in the mid-secretory eutopic endometrium of patients with AM compared with that in the control group. This decrease in LEF1 expression corresponded with reduced levels of the decidualization markers IGFBP1 and PRL, as well as the endometrial receptivity markers HOXA10 and LIF. These findings suggest that decreased LEF1 expression may play a crucial role in the inadequate decidualization and decline in endometrial receptivity observed in patients with AM.

To explore the downstream effectors of LEF1 that influence decidualization, we conducted a transcriptional profiling analysis in T-HESCs following shRNA-mediated knockdown of LEF1. Our analysis revealed 563 DEGs, among which IL-11 emerged as a key player in the decidualization process (Menkhorst et al. 2009; Karpovich et al. 2005; Paiva et al. 2009; Sonderegger et al. 2011). During embryo implantation, the uterine stromal cells exhibit high expression of IL-11, which initiates and maintains decidualization by activating the signaling transducer and transcription activator 3 and suppressor of cytokine signaling 3 (Dimitriadis et al. 2006, 2007). Mice with IL-11 gene knockout or those treated with an IL-11 antagonist display infertility characterized by impaired stromal cell differentiation and implantation failure (Agthe et al. 2017). Additionally, IL-11Ra-deficient mice exhibit defects in decidualization and alterations in the extracellular matrix (Bilinski et al. 1998), indicating that IL-11 regulates the proper progression of decidualization by modulating the uterine extracellular matrix. These findings are consistent with our current results.

Here, we observed a downregulation of IL-11 expression in the endometrium of patients with AM. To investigate the role of IL-11 further, we employed shRNA-mediated knockdown and overexpression techniques, which confirmed that IL-11 expression was positively regulated by LEF1. As expected, we successfully rescued the inhibitory effects of LEF1 knockdown on decidualization by overexpressing IL-11. On the basis of these findings, we propose that LEF1 plays a crucial role in regulating endometrial decidualization by mediating IL-11 expression.

Implantation marks the initial physical and physiological interaction between an implantation-competent blastocyst and a receptive uterus (Zhang et al. 2013; Shekibi et al. 2022). The dialogue occurring at the molecular level, orchestrated by the cross-talk between the embryo and uterus, guides the establishment of successful implantation. During the preimplantation period, the endometrium is programmed to secrete various molecules with autocrine and paracrine functions, and embryos express receptors for these molecules and hormones, thereby acquiring competence for adhesion and invasion (Craciunas et al. 2019; Franasiak et al. 2022). While blastocysts naturally adhere to the endometrial epithelium, stromal cell interactions are crucial for the later stages of implantation, including trophoblast invasion and placental formation. To further investigate the relationship between LEF1/IL-11 and endometrial receptivity, we expanded our observations to a heterologous co-culture system of embryo implantation, which simulated the process of trophoblast invasion into the endometrial stroma during in vivo implantation (Carver et al. 2003; Mardon et al. 2007). We discovered that LEF1/IL-11-knockdown HESCs displayed a marked deficiency in supporting trophoblast outgrowth, whereas HESCs with LEF1-knockdown that subsequently overexpressed IL-11 significantly rescued trophoblast outgrowth. This indicates that the reduced expression of LEF1/IL-11 during the mid-secretory phase of the endometrium is detrimental to embryo invasion and implantation. To gain a more thorough, in-depth understanding, we used a murine model of adenomyosis for fertility assessment, focusing on endometrial decidualization and receptivity during early implantation. Consistent with existing research, our study found significant uterine underdevelopment and a notable decrease in embryo implantation sites in adenomyosis mice. Our research also confirmed a reduction in LEF1 expression in these mice, indicative of impaired endometrial decidualization and reduced receptivity. Importantly, administering IL-11 improved these conditions, highlighting its potential as a therapeutic intervention for the fertility challenges in adenomyosis.

However, decreased endometrial receptivity is not the sole cause of adenomyosis-associated infertility. Other mechanisms may also contribute. Impaired sperm transport due to abnormal uterine contractility has been suggested, with altered myometrial contractility potentially hindering sperm motility and facilitating sperm pooling (Kissler et al. 2007). Stromal cell abnormalities, leading to defective trophoblast invasion and impaired placental development, may also play a role (Stratopoulou et al. 2024). This is especially relevant as adenomyosis is more commonly associated with early miscarriage rather than implantation failure, indicating that infertility in these patients is multifactorial. Thus, while endometrial receptivity is crucial, factors such as impaired sperm transport and stromal cell dysfunction must also be considered as contributors to adenomyosis-related infertility.

Our findings pave the way for a more profound understanding of the molecular mechanisms involved in AM and potentially unlock new paths for diagnostic and therapeutic strategies. We anticipate that our results will elucidate the pathological mechanism whereby reduced expression of LEF1 in the endometrial stromal cells of patients with AM leads to insufficient decidualization. This study holds promise for the discovery of new markers and interventional targets for the assessment of endometrial receptivity in patients with AM. Furthermore, this research has significant scientific merit in unveiling the regulatory mechanisms of endometrial receptivity and holds crucial clinical applicability in the early diagnosis and treatment of reduced endometrial receptivity in patients with AM, as well as in enhancing in vitro fertilization outcomes.

## Electronic supplementary material

Below is the link to the electronic supplementary material.

Supplementary Material 1

## Data Availability

The raw data generated for this study have been deposited in the NCBI Sequence Read Archive (SRA) under accession code PRJNA1188457.
