# Supplementary material for: Decreased expression of LEF1 caused defective decidualization by inhibiting IL-11 expression in patients with adenomyosis
Source: Mol Med. 2025 Jan 10;31:10. doi: 10.1186/s10020-024-01054-9 (PMC11720350; doi:10.1186/s10020-024-01054-9)
Supplement: Supplementary file 1 — Supplementary Material 1 [file 10020_2024_1054_MOESM1_ESM.docx]

**Supplementary** **Figure 1.** Flowchart of this research.


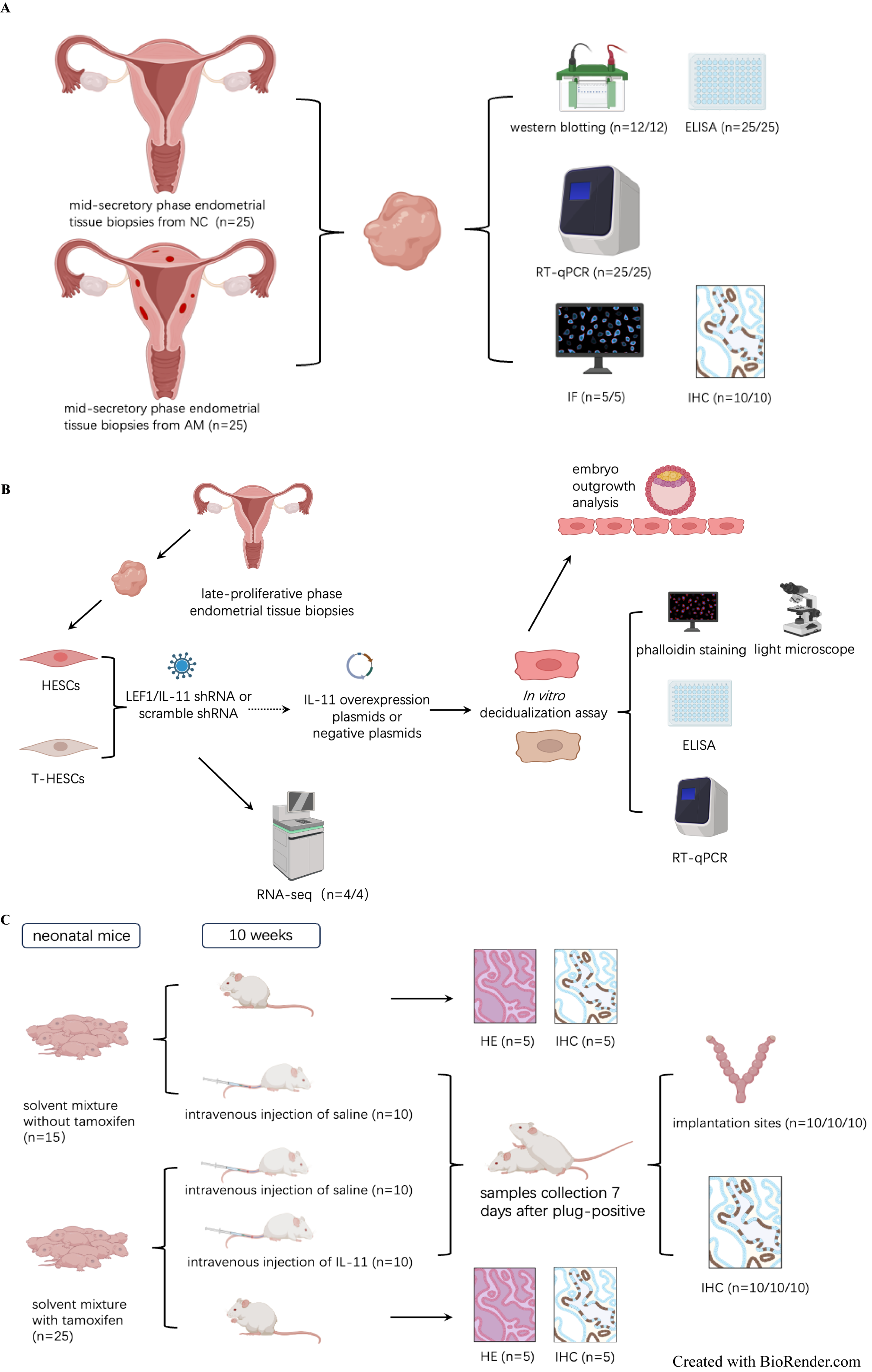


(A) *In vivo* experiments; (B) Functional experiments; (C) Mouse Model Experiments. AM, adenomyosis; ELISA, enzyme-linked immunosorbent assay; RT-qPCR, quantitative real-time polymerase chain reaction; IF, Immunofluorescence; IHC, Immunohistochemical staining; HESCs, human endometrial stromal cells; T-HESCs, TERT-immortalized Human Endometrial Stromal Cells.

**Supplementary Table 1.** Primers in this research.

| Gene | Sense primer (5′→3′) | Antisense primer (5′→3′) |
| --- | --- | --- |
| *LEF1* | ACAGATCACCCCACCTCTTG | TGAGGCTTCACGTGCATTAG |
| *PRL* | AGGAGCAAGCCCAACAGATG | TACTTCCGTGACCAGATGATACAG |
| *IGFBP1* | CCCAGAGAGCACGGAGATAAC | GGTGACATGGAGAGCCTTCG |
| *IL-11* | AGGTGGTCCTTCCCTAAAGACTCTG | CAAGAGCTGTAAACGGCGGAGTAG |
| *CEBPG* | ACTCCAGGGGTGAACGGAAT | CATGGGCGAACTCTTTTTGCT |
| *SMAD3* | TGGACGCAGGTTCTCCAAAC | CCGGCTCGCAGTAGGTAAC |
| *FGF22* | GGGAGCGCATCGAAGAGAAC | CTGTGAGGCGTAGGTGTTGTG |
| *BMP4* | ATGATTCCTGGTAACCGAATGC | CCCCGTCTCAGGTATCAAACT |
| *VEGFC* | GAGGAGCAGTTACGGTCTGTG | TCCTTTCCTTAGCTGACACTTGT |
| *FGF1* | GCCCTGACCGAGAAGTTTAATC | CCCCGTTGCTACAGTAGAGG |
| *GAPDH* | TGACTTCAACAGCGACACCCA | CACCCTGTTGCTGTAGCCAAA |
| *IL-11*(ChIP) | AGAGACACACCAGAGAAGCA | AAGTCCCTCCTGCTGTCTTC |

**Supplementary Table 2.** Antibodies in this research.

| **Antibody** | **Antibody Dilutions** | **Catalog** | **Application** |
| --- | --- | --- | --- |
| LEF1 rabbit mAb | 1: 1000 | Abcam, ab137872 | WB |
| HOXA10 goat pAb | 1: 500 | Abcam, ab191470 | WB |
| LIF rat mAb | 1: 1000 | Abcam, ab138002 | WB |
| GAPDH rabbit mAb | 1: 2000 | CST, #5174 | WB |
| Lamin B1 rabbit pAb | 1: 5000 | Proteintech, 12987-1-AP | WB |
| PRL rabbit mAb | 1: 1000 | Abcam, ab188229 | WB |
| IGFBP1 rabbit mAb | 1: 2000 | Abcam, ab180948 | WB |
| IL-11 rabbit mAb | 1: 500 | Abcam, ab187167 | WB |
| rabbit IgG, HRP-linked | 1: 1000 | Beyotime, A0208 | WB |
| rat IgG, HRP-linked | 1: 1000 | Beyotime, A0192 | WB |
| goat IgG, HRP-linked | 1: 500 | Beyotime, A0181 | WB |
| LEF1 rabbit mAb | 1: 100 | Abcam, ab137872 | IF |
| Vimentin mice mAb | 1: 200 | Abcam, ab8978 | IF |
| Cytokeratin 7 mice mAb | 1: 200 | Abcam, ab9021 | IF |
| Rabbit IgG (Alexa Fluor^@^488) | 1: 500 | Abcam, ab150077 | IF |
| Mouse IgG (Alexa Fluor 594) | 1: 500 | Invitrogen, A11005 | IF |
| LEF1 rabbit mAb | 1: 100 | Abcam, ab137872 | IHC |
| IL-11 rabbit mAb | 1: 100 | Abcam, ab187167 | IHC |
| HAND2 rabbit mAb | 1: 500 | Abcam, ab200040 | IHC |
| LIF rat mAb | 1: 200 | Abcam, ab138002 | IHC |
| Recombinant rabbit IgG | - | Abcam, ab172730 | IHC |
| Recombinant rat IgG | - | Yeasen, 36114ES60 | IHC |
| rabbit IgG, HRP-linked | 1: 1000 | Beyotime, A0208 | IHC |
| rat IgG, HRP-linked | 1: 1000 | Beyotime, A0192 | IHC |
| LEF1 rabbit mAb | 5μg | Abcam, ab137872 | ChIP |
| Rabbit IgG control pAb | 5μg | Proteintech, 30000-0-AP | ChIP |

**Supplementary Table 3.** shRNA Sequence (5’-3’) in this research.

| shRNA | Sequence (5’-3’) |
| --- | --- |
| NC (scramble) | TTCTCCGAACGTGTCACGTAA |
| *LEF1*-sh1 | GCACGGAAAGAAAGACAGCTA |
| *LEF1*-sh2 | CCATCAGATGTCAACTCCAAA |
| *LEF1*-sh3 | CGACCCATACATGTCAAATGGATCT |
| *IL-11*-sh1 | CGTGCTCCTGACCCGCTCTCT |
| *IL-11*-sh2 | TGCACAGCTGAGGGACAAATT |
| *IL-11*-sh3 | CCTACTGTCCTACCTGCGGCA |
